# Hearty Suppers

**Published:** 1877-10

**Authors:** 


**HEARTY SUPPERS.**—A case was recently stated in this JOURNAL, in which a clergyman rode from breakfast until night, without eating any thing. Weary and hungry, he ate a very hearty meal and retired to bed. During the night he was taken ill, fell at once into a stupor, and in that condition died next day. In another case, a man came into the hospital with both shoulders disjointed, the result of a hearty late supper, as there explained. In another instance, under our own observation, a person in as apparent good health as at any time during the fifty years preceding, was attacked with lung-fever, in consequence of hearty eating in the latter part of the afternoon, and died in three days. Usually, late and hearty suppers cause diarrhea, cholera, cramp-colic, and similar forms of disease, but in many cases their ill-effects are manifested in ways little suspected; hence they often get off with less than their share of blame. It is useful, therefore, to give some of their more uncommon results, this may lead a few of the wiser sort to adopt from principle, as a wise precaution, the safe, advantageous and rational practice of eating nothing later than the 6 P. M. meal, beyond a piece of cold bread and butter, adding, perhaps, not a glass of cold water, but what is better, a single teacupful of any hot drink. Lung-fever, inflammation of the lungs, and pneumonia, mean precisely the same thing, "Lung-Fever" being plain old Anglo-Saxon. The patient ate very heartily indeed, late in the after-part of the day, and waked up in the night with a severe chill, which shook the body like an aspen, and lasted for more than an hour; then came vomiting of bile, and looseness of bowels, and a little cough; these all suddenly ceased, and the patient sank rapidly into the grave, at the age of seventy-five. The lungs of this person had been so weak at the early age of twenty-two, that it was freely prophesied, that life could not last another year. When any thing is eaten, extra blood and heat go to the stomach to carry on the work of digestion, and this process ceases the instant the temperature is below nature's standard. An extra meal requires extra digestive power and extra heat; the blood is called in from the outposts, and so is the heat, to assist the stomach in its unusual labor; that leaves the surface, the skin, the feet, the fingers, cold. Has the reader never felt chills run over the body in getting up from a hearty meal? A greater degree of that would be a "regular shake." Now the lungs are in direct sympathy with the skin, hence the chill of the skin is often transferred to the lungs. In the case in question, the effects of the chill fell on the lungs, they being the weaker part, the chill of the skin drove all the blood inwards and congested it, heaped it on the incapable, the weak, the helpless lungs; as proof, the patient spat blood, a mouthful or less. It was nature's effort to get rid of the terrible load; if she had been able to clear herself of half a tea-cupful or more, the patient would have been saved, but there was not constitutional strength enough to make the effort; the clockwork of life had run so long, that all its wheels had pretty nearly worn out together, so that there was not power sufficient to overcome the obstacle of a pin-head, as it were. The main lesson of the article is, that eating heartily, late in the day, is always hurtful, sometimes dangerous and even fatal. And further, that safety in the old and the feeble consists in habitually guarding against even slight exposures, slight irregularities, and slight changes in any of the habits and practices of life, eating and drinking and exercising in the greatest moderation, being systematic and uniform in all things.

